# Multiple venous thromboembolisms in a pregnant patient carrying a novel mutation in *SERPINC1* (p.M313T) that causes a transient antithrombin deficiency: a case report

**DOI:** 10.1186/s12959-023-00571-7

**Published:** 2023-12-13

**Authors:** Yuwen Huang, Yinling Wang, Xiaoli Wang, Jue Liu, Bing Luo, Yuanmei Gao

**Affiliations:** 1 Department of Respiratory and Critical Care Medicine, The Third Affiliated Hospital of Guangzhou Medical University, Guangzhou, China; 2 Department of Critical Care Medicine, The Third Affiliated Hospital of Guangzhou Medical University, Guangzhou, China; 3 Maternal and Child Office, The Third Affiliated Hospital of Guangzhou Medical University, Guangzhou, China; 4 Medical Imaging Department, The Third Affiliated Hospital of Guangzhou Medical University, Guangzhou, China; 5 Blood Transfusion Department, The Third Affiliated Hospital of Guangzhou Medical University, Guangzhou, China

**Keywords:** Congenital antithrombin deficiency, Gene mutation, Genetic analysis, Pregnant, Transient deficiency, Venous thromboembolism

## Abstract

**Background:**

Congenital antithrombin deficiency is an autosomal dominant disease that results in deep venous thrombosis and pulmonary embolism, which is mainly caused by mutations in the antithrombin gene (*SERPINC1*). Since *SERPINC1* is highly susceptible to alterations, severe structural and functional changes that promote thrombosis may occur. Clinical presentations vary from different alterations. We report a pregnant case with novel mutation in *SERPINC1* presenting transient antithrombin deficiency and multiple venous thromboembolisms.

**Case presentation:**

We report a case of a 36-year-old pregnant patient who was diagnosed with congenital antithrombin deficiency for carrying a novel heterozygous mutation, NM_000488:exon5:c.T9 38 C:p. M313T in *SERPINC1* presenting transient antithrombin deficiency and multiple venous thromboembolisms. Thrombolytic with alteplase and anticoagulant therapies with low-molecular-weight heparin and warfarin were administrated. After confirming the genetic analysis and the termination of pregnancy, rivaroxaban was administrated, and the thrombosis reduced.

**Conclusions:**

Our study enriched the mutation database of *SERPINC1* gene, provided some new theoretical basis for gene diagnosis and genetic counseling of patients with transient antithrombin deficiency. While it still needs for subsequent exploration of molecular pathogenesis.

## Background

Venous thromboembolism (VTE) is an acknowledged multifactorial disease that contributes significant burden on health and survival, encompassing deep vein thrombosis (DVT) and pulmonary embolism (PE) [1, 2]. The pathogenesis of VTE results from hereditary and acquired risk factors. Infection, pregnancy, surgery, trauma, sedentary status, and cancer are considered acquired risk factors for VTE that may trigger the occurrence of VTE on an inherited thrombotic terrain. Studies have demonstrated that genetic factors are responsible for more than 60% of the common thrombotic susceptibilities [3, 4].

Antithrombin (AT) secreted by the liver is the most important physiological anticoagulant in the plasma [4]. A pentasaccharide binding site and an active reaction center are two distinct domains on the molecule which primarily involved in the mechanism of the inhibitory function of AT. Due to contain specific pentasaccharide sequences, AT activity is greatly accelerated in the presence of heparin or heparan sulfate. The presence of heparin results in a conformational change, the reactive site is exposed, and the target protease is then cleaved by the scissile bond of the reactive site and captured by the inhibitor [5]. Deficiency increases the risk of venous thrombosis by 5–50 fold because of the unique anticoagulant mechanism and extensive anticoagulant activity of AT [6]. The prevalence of AT deficiency accounts for 1–5% in patients with VTE [7]. AT deficiency can be categorized as either hereditary or secondary [6].

Congenital antithrombin deficiency (CAD) is an autosomal dominant disease [8, 9]. The clinical presentations of CAD include DVT and PE [9]. The incidence rate of CAD is 1 in 500–5000 adults, in whom the risk of VTE increases 20-40fold as compared with the general population [8, 10]. Among the selected Serbian patients, women with pregnancy-related VTE predominantly had AT deficiency, which accounts for 6% [9]. CAD is mainly caused by mutations in the AT gene (*SERPINC1*) [6].

*SERPINC1* located on chromosome 1 q23.1–25, is 13.5 kb in length, and contains seven exons and six introns [10]. *SERPINC1* is highly susceptible to alterations, not only small insertions or deletions, gross gene defects, but also small changes in its nucleotide sequence may contribute to severe structural and functional changes of AT, which may promote deleterious consequences leading to AT deficiency [6, 11]. A high percentage of cases with AT deficiency (70–80%) are explained by mutations or deletions affecting exons of *SERPINC1* [12]. Most clinical cases are heterozygous because it is difficult for homozygotes to survive as has been proven in mouse models [13]. The *SERPINC1* mutation profile is highly heterogeneous, with 529 *SERPINC1* mutations recorded in the HGMD as of January 2023. Among them, 338 different gene variations in *SERPINC1* identified in patient with AT deficiency. (https://www.hgmd.cf.ac.uk/ac/index.php gene = *SERPINC1*).

However, there are few studies evaluating the impact of a well characterized CAD in pregnancy [8, 9]. To enhance the understanding of CAD with novel mutation in *SERPINC1*, we report the case of a pregnant patient who was diagnosed with multiple VTE and CAD determined by a novel mutation in the *SERPINC1* gene.

### Laboratory measurement

The AT activity was determined by chromogenic methods based on factor Xa inhibition. The reference range for AT activity was from 83 to 128%.

## Case presentation

A 36-year-old pregnant Chinese patient who was previously healthy complained of dyspnea for three weeks and deterioration for five hours in the second trimester of pregnancy (22 weeks of gestation) and was admitted to local hospital. White blood cell counts and neutrophil percentage increased. Chest computed tomography confirmed lung infection and pleural effusion formation. Arterial blood gas analysis revealed hypoxemia (PO_2,_ 62mmHg, SpO_2,_ 93%). D-dimer, 26,210 ng/mL↑. Limb vein ultrasound confirmed right cephalic vein thrombosis. Echocardiography showed that thrombus formation from right ventricle to right ventricular outflow tract, from pulmonary artery bifurcation to left pulmonary artery and proximal right pulmonary artery. Mild tricuspid regurgitation. Mild pulmonary hypertension (46mmHg). Enlargement of the right heart. Pulmonary artery computed tomography angiography (CTPA) confirmed right ventricular and bilateral pulmonary artery thrombosis. The patient was diagnosed with PE, pulmonary hypertension and pneumonia. She was administrated with heparin (25,000 IU per day), 50 mg alteplase thrombolysis, antibiotic (ceftriaxone) and transferred to the Intensive Care Department of our hospital. BMI was 22.3 kg/m^2^. The patient doesn’t smoke, or take hormone drugs. The families had no VTE phenotypes and no family history of venous thrombosis. Self-tested SARS-CoV-2 antigen was positive three weeks ago when the dyspnea appeared.

Laboratory tests were as follow: the activity of AT, 53% (Reference range:83–128%) (Fig. 4); D-dimer, 4132 ng/ml↑; Biomarkers of myocardial injury: high sensitivity troponin T, 71.69 ng/L↑ (0–14 ng/L), myoglobin and creatine kinase isoenzymes were not significantly abnormal. Thrombophilia screening: Protein C, Protein S activity, and anti-cardiolipin antibody combinations were not significantly abnormal. Anti-nuclear antibody and antinuclear antibody profiles, and anti-neutrophil cytoplasmic antibody were negative; Lipids: Total cholesterol 5.46mmol/L↑, triglycerides 1.82mmol/L↑,HDL cholesterol 0.61 mmol/L↓, LDL cholesterol 4.2 mmol/L↑, ApoA1 0.85 g/L↓, ApoB 1.15 g/L, lipoprotein (a) 36 mg/L; Infective index: Blood, phlegm culture, SARS-CoV-2 RNA was negative; Interleukin-6 12.362 pg/ml↑(0-5.4pg/ml);General item: liver and kidney function, electrolytes, thyroid function, stool and urine routine, HbA1c did not show any significant abnormalities. Genetic analysis: a novel heterozygous mutation (NM_000488: exon5: c. T938C: p.M313T) in *SERPINC1*. FII, V and other thrombogenic gene were normal, to sequencing of F5, F7 and other genes involved in thrombosis (indicate the genes sequenced) revealed no pathogenic variants.

**Fig. 1:**
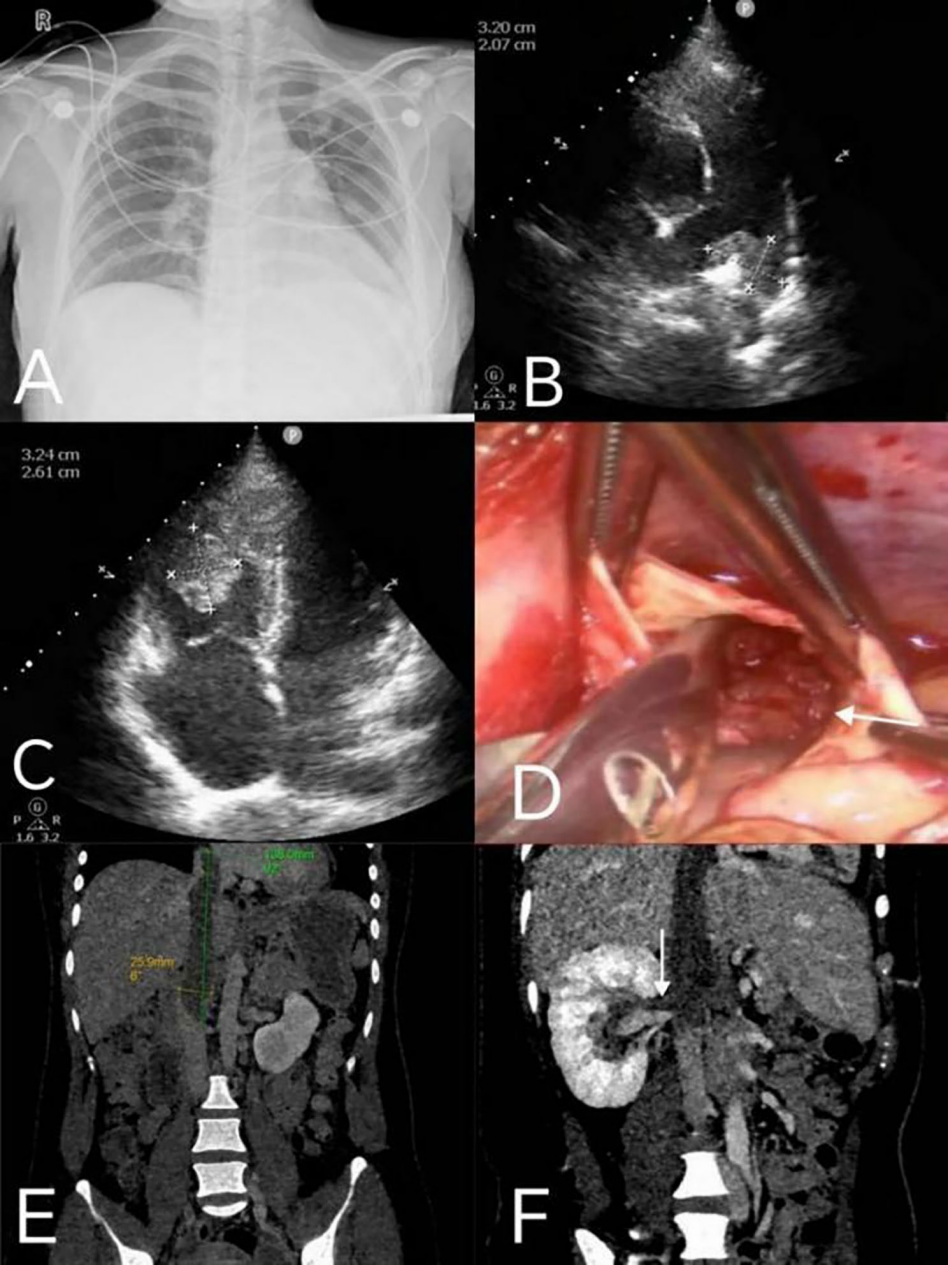
The results of imaging examination. (**A**) Chest radiography when first admission (**B**) Echocardiography revealed thrombosis of the pulmonary artery when first admission (**C**) Echocardiography revealed thrombosis in the right ventricle when first admission (**D**) Thoracoscopic intracardiac and pulmonary thrombus removal using extracorporeal circulation (**E**) Main abdominal vein computed tomography angiography shows that embolism in the cava when third admission (**F**) Main abdominal vein computed tomography angiography shows that embolism in the renal veins when third admission

**Fig. 2:**
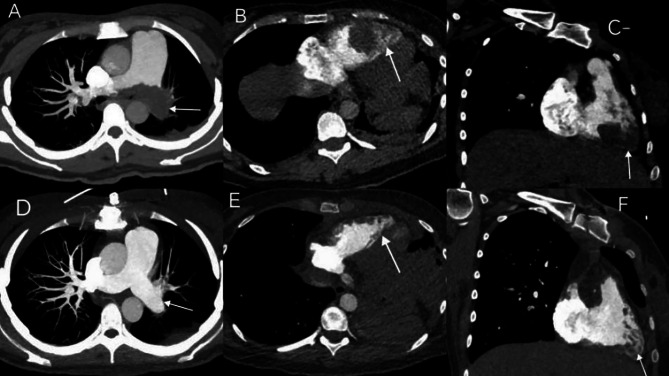
CTPA before and after operation. (**A-C**) CTPA scan on first admission in our hospital (**D-F**) CTPA scan at post-operation

After the patient was transferred to our hospital, electrocardiogram results were that right electrical axis deviation, incomplete right bundle branch block. Chest radiography revealed an enlarged cardiac shadow, a cardiothoracic ratio of approximately 0.56, and a distended pulmonary artery segment (Fig. 1A). CTPA revealed multiple embolisms of bilateral main pulmonary artery, and branches, mainly in the left lower pulmonary artery segments (Fig. 2A), and the right ventricular filling defect was considered for embolization (Fig. 2B-C). Echocardiography suggested thrombosis in the pulmonary artery and right ventricle (Fig. 1B-C). Venous ultrasonography of both the upper and lower extremities was normal, iliac vein couldn’t be seen because of fetal occlusion. Echocardiography revealed a massive thrombus in the right ventricle (32 × 26 mm). Considering the rapid ventricular blood flow rate, the thrombus would be in high risk of shedding. When the huge thrombus shed, it may obstruct the main pulmonary artery, with high risk of a sudden death. After a multidisciplinary evaluation, the patient met the surgical indications and underwent thoracoscopic intracardiac and pulmonary thrombus removal via extracorporeal circulation (Fig. 1D). Postoperative CTPA showed the thrombus in the main pulmonary artery and multiple embolisms in the branches of the two pulmonary arteries have disappeared (Fig. 2D). The embolism in the right ventricle reduced after surgery (Fig. 2E-F). During hospitalization, based on the weight of the patient (60 kg), she was administrated with 6000IU low-molecular-weight heparin (LMWH) per 12 h for anticoagulation. The patient was discharged when her condition was stable.

**Fig. 3:**
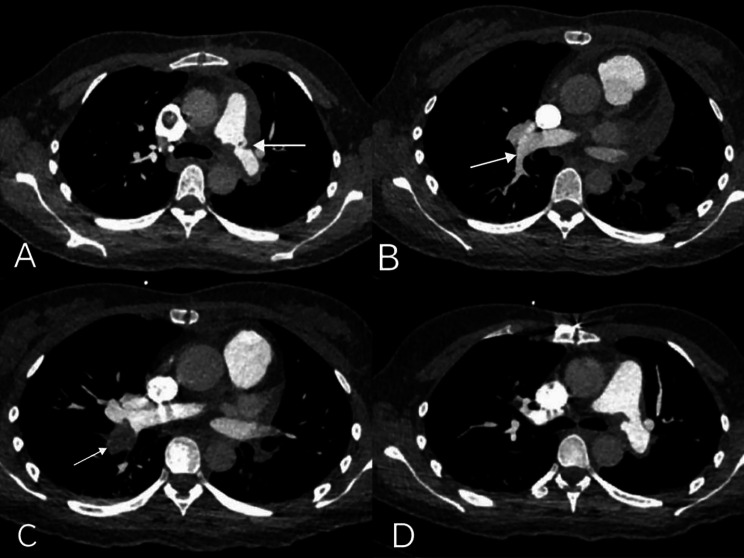
CTPA before and after rivaroxaban. (**A**) CTPA review showing a more advanced embolism in the left pulmonary artery on second admission (**B**) CTPA review showing a normal right lower pulmonary artery on second admission (**C**) Thrombosis in the right lower pulmonary artery on third admission (**D**) Reduced thrombus after switching to rivaroxaban for anticoagulation

One month after surgery, the patient was readmitted to terminate pregnancy for diseases of the fetal nervous system. The patient continued to be administrated with LMWH (5000 IU per 12 h, 54 kg) after discharge. The patient had no discomfort and her vital signs were stable when admission. While, D-dimer, 1608 ng/ml↑and a review of the CTPA at that time showed that the embolism in the left pulmonary artery was larger than before, new embolism was found in superior and inferior vena cava (Fig. 3A-B). Considering the poor effectiveness of LMWH, we changed it into oral warfarin for anticoagulation two days after delivery (2023/03/10). After discarding an acquired deficiency, [14] we performed a genetic analysis of thrombophilia for suspected CAD. A month after discharge, the dose of warfarin was increased from 1.25 mg to 3.75 mg once a day based on international normalized ratio (INR) monitoring results in local hospital. On follow-up, the INR of the coagulation test haven’t reached the target (1.04–1.41).

A month after the second discharge from our hospital, the patient made third admission with complaining about colic of left lower abdomen and left lower back. Physical examination revealed deep tenderness near the umbilical area of the left abdomen and pain perception in the left renal region. Laboratory examination showed AT activity, 88%. D-dimer, 5363 ng/ml↑. Genetic analysis revealed a novel heterozygous mutation (NM_000488: exon5: c. T9 38 C: p. M313T) in *SERPINC1*, with an allele frequency of 0.0003 in East Asian populations in the ExAC database. According to analysis, the p.M313T mutation is rare and has a low incidence in the population. While the predicted result of PolyPhen2 mutation is “Probably Damaging,” the predicted result of SIFT mutation is “Damaging,” both indicating that the mutation of *SERPINC1* gene is harmful. The patient’s son completed genetic testing which showed no mutation, while other family members refuse to complete it for financial or distant reasons. CTPA showed that the right pulmonary artery thrombosis wasn’t worsen. Main abdominal vein computed tomography angiography revealed multiple thrombosis in inferior vena cava, superior mesenteric vein, half azygos vein, left renal vein, proximal right renal vein, and bilateral internal iliac vein were found (Fig. 1E-F). We change into rivaroxaban (15 mg twice a day) for anticoagulation. Three days after rivaroxaban administrated, and before discharge, the thrombus remained stable (Fig. 3D). The D-dimer level decreased to 1002 ng/ml, and AT activity was 108%. After discharge, the patient regularly took rivaroxaban and was reexamined on time in local hospital with echocardiography to monitor changes of thrombus. It indicated thrombus in right ventricle is shrinking. CTPA indicated that the main pulmonary artery and right pulmonary artery thrombus had disappeared, the inferior vena cava, right atrium, and right ventricle were partially reduced. Abnormalities on venous ultrasound of either lower limb were not present. Laboratory tests indicated D-dimer was 1160 ng/ml. Based on these results, the patient’s condition improved, indicating that rivaroxaban was effective.

## Discussion and conclusions

We report a pregnant patient presenting with refractory and recurrent multiple VTE and transient AT deficiency. Genetic detection showed heterozygous mutation of *SERPINC1* (c.T938C) was identified in exon five. The variant is described in GnomAD with relatively high prevalence in Asian population (0.0006014). The patient went through recurrent and multiple VTE with therapeutic LMWH. After rivaroxaban treatment, no adverse events occurred more than one year follow-up. VTE is well-known as a multicausal disease influenced, the multiply inherited and acquired factors participated important role in the occurrence and prognosis of this disease [7].

**Fig. 4:**
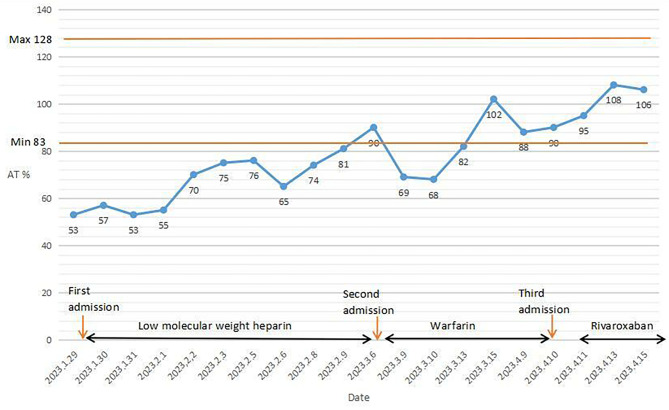
The timeline during the treatment in hospital and AT activity

According to previous study, there are many acquired causes of low AT% [14]. In this case, surgery, thrombosis and pregnancy were considered the causes of consuming leading to low AT%. During the first and second hospitalization, the pregnant patient not only had thrombosis, but also experienced thoracic surgery and induced surgery. Multiple acute events might result in the consumption of AT, which corresponds to low level of AT% (Fig. 4). According to existing studies, acute events and pregnant stage will consume not only AT, but also Protein C and Protein S [14]. While the level of Protein C and Protein S were normal in this case. Cambridge II variants, a predominant mutation in *SERPINC1*, the carriers of this mutation might loss a significant proportion of the native variant AT under mild generation of thrombin, with the consequent loss of anticoagulant capacity [15].Further studies are needed to determine whether the transient AT deficiency is due to multiple acute events depleting the AT, or insufficient AT secretion because of genetic mutations, or the patient of this mutation might loss a significant proportion of the native variant AT under mild generation of thrombin similar to Cambridge II variants under acute consumption. Only by functional assays might miss some patients with CAD, such as transient AT deficiency which was defined as transitory deficiency of AT and the possibility that the deleterious effect of pathological alterations is manifested only in certain conditions at certain times [16, 17]. In the third hospitalization, the patient was diagnosed with multiple venous thrombosis, while AT % were in normal range. Hence the structure of AT was suspected changed due to gene mutation. The external factors like pregnancy that may trigger such inactivating transitions of AT, while we still need further study to clarify it.

CAD is an established risk factor of VTE. It has been reported that up to 80% of CAD are caused by mutation of the AT gene (*SERPINC1*) [11]. Classically, AT deficiency are defined and divided into two types according to the ratio antigen/anticoagulant activity detected in plasma. Type I deficiency (quantity deficiency) was associated with nonsense, frameshift mutations causing premature stop codons, gross gene defects, and splicing mutations in *SERPINC1* and then prevents hepatic cells from the synthesis of AT. The pathogenesis of type II (quality deficiency) is missense mutations [12, 18]. Within type II deficiency, three sub-types can be distinguished according to the functional defect, including II a or reactive site, IIb or heparin binding site and II c or pleiotropic [12]. While some 40 different missense mutations have now been found to lead to a severe type I deficiency often related with pleiotropic consequences, which illustrated that these variants raised the likelihood that the unusually severe disease resulted from an aberrant conformational change rather than just the loss of activity [19]. AT is a structurally related globular protein, a typical three-dimensional structure, that consists of nine α-helices and three β-sheets [19]. It contains a primary feature that reactive center loop of circulating native AT is partially inserted into the body of the molecule, at the same time obscuring the arginine, a key amino acid determining the specificity of inhibition to thrombin and factor Xa. The modification of just a single amino acid in these sensitive regions can disturb the network of interactions in the whole molecule, resulting in overall on formational changes that can affect all the functions of the molecule, including its inhibitory activity and its heparin binding affinity [19]. Picard, V. reported that some substitutions, p.F306L (F274L), p.L317S (L285S), p.P318L (P286L), involved a highly conserved amino acid. These substitutions were non conservative, strongly suggesting that the change has a major impact on protein synthesis and/or folding, resulting in the absence of secretion [20]. Picard et al. reported another two mutations, p. Leu285Ser and p. Pro286Leu, located in the s3B, which were also associated with a type I AT deficiency. They indicated that these two nonconservative substitutions had a significant effect on protein synthesis and/or folding, resulting in the absence of secretion [21]. Liu, S. have concluded that this heterozygous missense mutation c.938T > C (p.Met281Thr) is responsible for the type I AT deficiency in this Chinese family. In our case, since Met313 and above conserved amino acids are all located in the same secondary structure region s3B and adjacent, the conservation of amino acids plays a vital role in the function and structure of AT molecules. We speculated that the pathogenic mechanism of Met313Thr was the same as those. The missense change resulting in the affected residue in s3B and its conservation in the serpin superfamily may suggest a structural relevance which indicates that the generation of a new non-conserved amino acid may affect the intramolecular disulfide bonds and probably affects a correct folding potentially causing a type I deficiency. To further investigate the pathogenic characteristics of this mutation, we need to perform not only AT antigen but also bioinformatics and structural analyses of AT.

Apart from genetic mutation, Corona Virus Disease 2019 (COVID-19) and pregnancy are risk factors of thrombosis. While, COVID-19 is associated with a high prevalence of VTE, particularly PE. It was observed that thrombotic lesions in COVID-19 patients were found to be in lung parenchyma affected by COVID-19 in previous study. It is suggested that COVID-19 associated PE represents in situ immune-thrombosis rather than VTE, resulting from severe cytokine storm induced by SARS-Cov-2, which characterized as the absence of DVT, multiple thrombi in small to mid-sized pulmonary arteries, lower total clot burden [22, 23]. In this case, the symptoms of COVID-19 were not typical, the patient got no fever and the cytokine were normal. The site of pulmonary thrombosis was not related to lung parenchyma affected by COVID-19. CTPA indicated that the high burden clot was found in the right ventricle and main pulmonary artery. There still need further studies to clarify the mechanism. Even though iliac vein, the most common site of venous thrombosis in pregnant women, [24] it couldn’t be seen by ultrasound because the gravid uterus obscured the pelvic veins. The incidence of COVID-19 related thrombosis might not be used to explain the patient’s condition. Approximately 60% of the thrombotic events in AT-deficient individuals are caused by pregnancy [6]. Patient was pregnant in the second trimester of pregnancy (22 weeks of gestation). Because the lack of typical symptoms during the onset of COVID-19, considering dyspnea might be caused by the decrease of lung activity due to the enlarged uterus, and avoiding radiation to the fetus. When the patient found that the novel coronavirus antigen was positive, she did not seek medical treatment in time, delaying the best time for treatment. Secondly, childbirth and surgery result in hypercoagulability and vascular damage, which are also important acquired risk factors for VTE. Previous studies have indicated that AT concentrate can be selected at particular moment (such as during surgery or childbirth) for AT deficiency patients with IIb (poor heparin response) [25]. Therefore, for pregnant patients with CAD, it is necessary to complete an individual assessment for VTE risk. In addition, the type and extent of AT deficiency, the patient and family history, and additional preexisting risk factors (e.g., age, BMI, and comorbidities) should be considered. Furthermore, specific pregnancy- and postpartum-related risk factors and patient’s preferences should be taken into consideration.

In clinical practice, AT deficiency faces the dilemma of underestimation, including the transient AT deficiency. Traditional diagnostic approaches born with false negatives in thrombophilia. Our study enriched the mutation database of *SERPINC1* gene, provided some new theoretical basis for gene diagnosis and genetic counseling of patients with transient AT deficiency. While it still needs for subsequent exploration of molecular pathogenesis. There are also some limitations in this case. Due to laboratory techniques limitations, antigen levels and heparin affinity were not completed. The family analysis was not completed. Future work should be undertaken to explore the molecular bases of this mutation.

## Data Availability

Not applicable.

## Abbreviations

VTE: Venous thromboembolism
DVT: Deep vein thrombosis
PE: Pulmonary embolism
AT: Antithrombin
CAD: Congenital antithrombin deficiency
HGMD: Human Gene Mutation Database
CTPA: Pulmonary artery computed tomography angiography
LMWH: Low-molecular-weight heparin
INR: International normalized ratio
COVID-19: Corona Virus Disease 2019
